# Geriatric Assessment of Older Adults With Cancer During Acute Hospitalizations: Challenges and Opportunities

**DOI:** 10.7759/cureus.44940

**Published:** 2023-09-09

**Authors:** Helena Guedes, Joana Marinho, Ema Neto, Sandra Custódio, António Costa, Rafaela Veríssimo

**Affiliations:** 1 Oncology, Centro Hospitalar Vila Nova de Gaia/Espinho, Vila Nova de Gaia, PRT; 2 Internal Medicine / Geriatrics, Centro Hospitalar Vila Nova de Gaia/Espinho, Vila Nova de Gaia, PRT; 3 Internal Medicina / Geriatrics, Centro Hospitalar Vila Nova de Gaia/Espinho, Vila Nova de Gaia, PRT

**Keywords:** oncology, clinical frailty, older adult, comprehensive geriatric assessment, geriatric syndromes

## Abstract

Introduction

Geriatrics is a discipline that covers all adult healthcare, and oncology is no exception. The global geriatric assessment process plays a crucial role, impacting research, funding, resource allocation, as well as therapeutic decision-making. Thus, greater knowledge of the epidemiology of the frailty and functionality of elderly patients with cancer will allow for the development of a global care strategy. This study aimed to assess the prevalence of geriatric conditions in elderly cancer patients admitted to a medical geriatric unit following unplanned hospitalisation.

Methods

A retrospective, single-centre cohort study was conducted of patients aged ≥ 75 with an active oncological disease who were admitted to a geriatric medicine unit over a two-year period.

Results

A total of 65 patients were included. The median age was 85 (IQR 81-88), and 86% were aged ≥ 80. A moderate-to-high functional dependence was found: 67.7% on ≥ 3 basic activities of daily living (Katz ≥ D), with the majority classified as severely dependent based on the Barthel Index (mean 49.0 ± 33.7). Frailty was found in 90.7%. A high prevalence of geriatric syndromes was observed: malnutrition (84.6%), polypharmacy (64.6%), urinary incontinence (58.5%) and pressure ulcers (33.8%). The mortality rate was 36.9% during hospitalisation and 13.8%, 30 days post-discharge.

Conclusions

The study revealed a high prevalence of geriatric conditions, emphasising the importance of comprehensive assessment in managing elderly patients at different stages of the disease. This multidimensional and multidisciplinary approach optimises patient care throughout admission, hospitalisation and discharge.

## Introduction

Ageing is one of the strongest and most predictable risk factors for the development of cancer. The rapidly expanding pool of patients over the age of 65 both who are diagnosed with and who survive cancer is rising, as more than 60% of newly diagnosed cancer patients are aged 65 or older [1, 2].

Chronological age is often used for patient stratification in oncology as well as a criterion in randomised clinical trials. However, older adults with cancer comprise a heterogeneous population in which biological age and functional status often poorly correlate with chronological age alone. A Comprehensive Geriatric Assessment (CGA) of older adults with cancer before oncological treatment is recommended by the American Society of Clinical Oncology (ASCO) and International Society of Geriatric Oncology (SIOG) guidelines [3, 4].

The CGA is an important tool for the management of elderly cancer patients, as it collects information on geriatric conditions, identifies problems, and increases understanding of a person’s care needs with the development of an integrated treatment plan. In fact, CGA involves a systematic examination of domains in which elderly patients frequently experience deficits. This encompasses their functional capacity, mobility, cognitive function, emotional well-being, nutritional health, coexisting medical conditions, medication complexity, and the extent of their social network. By utilizing the information collected from CGA, we can estimate the extent of a patient's frailty, formulate an appropriate management strategy, and develop an integrated treatment plan [5, 6].

Frailty is a state of vulnerability to poor resolution of homeostasis following a stressor event, such as chemotherapy or hospitalisation. Hospitalisation is common among patients with advanced malignancies and is more likely among those who receive chemotherapy. Better knowledge of the epidemiology of frailty and functionality in older cancer patients during acute stressors such as hospitalisations could help drive a global cancer care strategy.

The main aim of this work was to evaluate the prevalence of geriatric syndromes and deficits in the CGA of older cancer patients after an unplanned admission to a geriatric unit of an internal medicine department.

## Materials and methods

A retrospective, single-centre observational cohort study was performed that included consecutive patients (≥75 years old) with histologically confirmed solid tumours diagnosed in the previous five years who were admitted to an internal medicine geriatric unit between January 2018 and December 2019. We performed a baseline assessment of patient and tumour characteristics and recorded the cause of hospital admission obtained from electronic medical records.

Geriatric conditions were evaluated at admission and included the following: comorbidities (Charlson Comorbidity Index), functional status (Katz and Barthel Index) [7-9], malnutrition (Nutritional Risk Screening - 2002) [10], polypharmacy (START/STOPP criteria) [11], cognitive impairment (Pfeiffer questionnaire) [12], common geriatric syndromes such as delirium (confusion assessment method) [13]; incontinence and pressure ulcers were also evaluated. Frailty was assessed using the Clinical Frailty Scale (CFS) [14]. In-hospital and 30-day mortalities were also calculated.

Statistical analysis was performed using SPSS software version 25.0. Normally distributed continuous variables were expressed as mean values and standard deviation (SD), and categorical variables were presented as absolute and relative frequencies. The study was undertaken with the explicit endorsement and consent of the health ethics committee of the institution wherein it was conducted (Approval Number: 82/2020).

## Results

A total of 65 patients were included. The majority (50.8%, n = 33) were male, the median age was 85 (IQR 81-88), and 86.0% were over 80 years old. The most frequently diagnosed tumours in this population were prostate cancer (16.9%, n=11) followed by colorectal cancer (15.4%, n=10). Most had an advanced-stage disease (stage III/IV: 50.8%, n=33). Only 16.9% (n=11) of the patients (n=11) were under cancer treatments, of which 72.7% (n=8) had palliative intent (Table 1).

**Table 1:** Patients and treatment characteristics IQR: Interquartile range *Kidney Cancer and Kaposi Sarcoma. ** Missing values.

	Total (n=65)	
Age (Median, IQR)	85 (81-88)	
Gender	n	%
Male	33	50.8
Female	32	49.2
Primary tumour location	n	%
Prostate	11	16.9
Colorectal	10	15.4
Hematological	9	13.8
Breast	9	13.8
Lung	7	10.8
Bladder	5	7.7
Skin	5	7.7
Brain	4	6.2
Hepatobiliary	3	4.6
Others*	2	3.0
Stage	n	%
I-II	29	44.6
III-IV	33	50.8
Without stage**	3	4.6
Treatment		
Patients in active treatment, n (%)	11 (16.9)	
Treatment	n	%
Curative	3	27.3
Palliative	8	72.7

Relative to comorbidities and functional status, the mean Charlson Comorbidity Index in this population was 8.2 ± 1.8. A high functional dependence was found, with 67.7% of patients being dependent for at least three basic activities of daily living (Katz ADL ≥ D) and 38.5% with severe dependence based on the Barthel Index (mean 49.0 ± 33.7) (Table 2).

**Table 2:** Comorbidities and functional, nutritional, and mental status evaluation on the admission day SD: standard deviation

	Total (n=65)				
Charlson comorbidity index, mean ± SD	8.2 ± 1.8				
Activities of Daily Living	n			%	
KATZ Index					
A	6			9.2	
B	7			10.8	
C	8			12.3	
D	10			15.4	
E	3			4.6	
F	11			16.9	
G	20			30.8	
Barthel Index, mean ± SD	49.0 ± 33.7				
Nutritional Status	n		%		
Nutritional Risk Screening- 2002					
≥3	55			84.6	
<3	10			15.4	
Polypharmacy	n		%		
≥5 medications	42			64.6	
Other Geriatric Syndromes	n		%		
Presence of pressure ulcers	22			33.8	
Urinary incontinence	38			58.5	
Mental Status	n	%			
Cognitive impairment	37			57.8	
Delirium	17			26.2	

Frailty was reported in 90.7% of patients on the CFS (CFS > 4), and in 70.7% of patients (n=46), their CFS was ≥ 6. Pressure ulcers were highly prevalent (33.8%). Cognitive impairment, including dementia and mild cognitive impairment at admission, was present in 57.8 %, and 26.2% developed delirium during hospitalisation. The main reason for hospital admission was infection (respiratory, urinary or skin) in 42.9% of patients (n=32), followed by disease-related symptom management (23.1%, n = 17). In 26.3% of patients (n=17), hospital admission was related to their disease and/or treatment complications. Of all patients, 36.9% (n = 24) died during hospitalisation. Regarding post-discharge care, 41.5% (n = 27) were discharged to their family homes and the remainder to a continuing care unit. The mortality rate at 30 days post-discharge was 13.8%. Readmission at 30 days was 12.2%.

## Discussion

Cancer patients are frequently admitted to hospital due to acute conditions or refractory symptoms [15]. In our study, a high percentage of geriatric syndromes was observed, underscoring the need for optimisation during hospitalisation and post-discharge. The Charlson Comorbidity Index demonstrated a high level of comorbidities. Regarding functional status, the Katz and Barthel indices highlighted a considerable degree of functional dependence, which might be explained by the median age of this patient population (85 years), which represents a very old population, but also accounts for the comorbidities. Specific interventions were carried out to enhance the patients’ general health based on the identification of CGA deficits: a tailored nutritional plan was established for each patient; the patients were also individually assessed at discharge using the STOPP and START criteria [11, 16]. This approach minimised iatrogenic harm and facilitated the initiation of appropriate therapy, consequently addressing the issue of polypharmacy.

For the few patients who were able to actively participate, tailored physical rehabilitation sessions were conducted in the hospital gym to improve physical function and mobility based on their individual capabilities. Comorbidity optimisation and social interventions during hospitalisation and after discharge were also performed. The elevated mortality rate in our cohort may be attributed to several contributing factors, such as the acute complications that motivated hospitalisation in patients with advanced stages of disease, old age and a high degree of functional dependence. Our findings highlight the importance of employing the CGA during patient hospitalisation. In the case of older cancer patients, regardless of the patient’s medical requirements or the team’s expertise, the CGA provides a comprehensive understanding of the complexity of the medical needs of the patient population and facilitates the provision of tailored care plans and the ability to address and anticipate geriatric syndromes more effectively [15]. The specific method used to conduct a CGA does not seem to significantly affect the outcomes as long as there is a pre-established intervention plan in case the team lacks expertise in addressing geriatric impairments [15]. In fact, studies have demonstrated that elderly medical patients do better when hospitalised in a geriatric ward than in other medical departments [17]. Although the population in this study had a high mortality rate, older patients who received CGA upon admission had a greater likelihood of surviving and residing in their own homes during follow-up [6]. The inclusion of CGA during hospitalisation enables patients to receive personalised treatment plans that optimise their care during hospitalisation and after discharge.

The study has limitations. It is a retrospective single-centre study with a small number of patients with different tumour types in various stages of disease. However, this is one of the first studies of its kind conducted in a multidisciplinary geriatric unit in our country that targets a very old cancer population.

## Conclusions

The study revealed a high prevalence of geriatric conditions, emphasising the importance of comprehensive assessment in managing elderly patients at different stages of cancer. With the average life expectancy on the rise, it is expected that the presented numbers will increase in the coming decades, which will have a significant impact on geriatric units and the need for greater proximity to oncology within these units. As a result, the authors advocate for a global geriatric approach that focuses on understanding the vulnerabilities of patients to reduce their frailty, as these factors play a crucial role in managing elderly cancer patients.
